# Use of a Novel Portable Non-powered Suction Device in Patients With Oropharyngeal Dysphagia During a Choking Emergency

**DOI:** 10.3389/fmed.2021.742734

**Published:** 2022-02-02

**Authors:** Matthew J. McKinley, Jennifer Deede, Brian Markowitz

**Affiliations:** ProHEALTH Care Associates, Lake Success, NY, United States

**Keywords:** choking, resuscitation, portable non-invasive non-powered suction device, dysphagia, oropharyngeal dysphagia, emergency, life saving

## Abstract

Choking remains a leading cause of accidental death and morbidity worldwide. Currently, there is no device to assist in the resuscitation of a choking victim when standard maneuvers fail. A novel portable non-powered suction device (LifeVac; LifeVac LLC, Nesconset, NY) has been developed and may have potential use in patients with oropharyngeal dysphagia who are at increased risk of choking. The device is FDA registered and distributed worldwide. This case series provides a summary of self-reported data regarding the use of the suction device in adult patients with oropharyngeal dysphagia during real-world choking emergencies recorded between January 2014 and July 2020. Over a 6-year monitoring period the device has been reported to be successful in the resuscitation of 38 out of 39 patients with oropharyngeal dysphagia during choking emergencies. Although the obstruction was removed with the device from the 39^th^ patient, resuscitation was not successful and he succumbed to his injuries. This portable, non-powered suction device may be useful in resuscitating patients with oropharyngeal dysphagia who are choking. The reported cases describe successful use of the device in real-world settings with minimal risk. Resuscitating patients with oropharyngeal dysphagia using this device may be a viable option when abdominal thrusts or back blows fail to resolve a choking emergency.

## Introduction

The swallowing process is a complicated orchestration of skeletal muscles, requiring rapid coordination (1). Numerous neurologic and musculoskeletal conditions can lead to oropharyngeal dysphagia, including stroke, Parkinson's disease, amyotrophic lateral sclerosis, and myasthenia gravis, which increase the risk of choking (2). Medical conditions affecting skeletal muscle coordination and strength can also cause oropharyngeal dysphagia, including polymyositis, and very young (children or toddlers) or old age. Certain medications can also increase the risk of oropharyngeal dysphagia (3).

**Figure 1:**
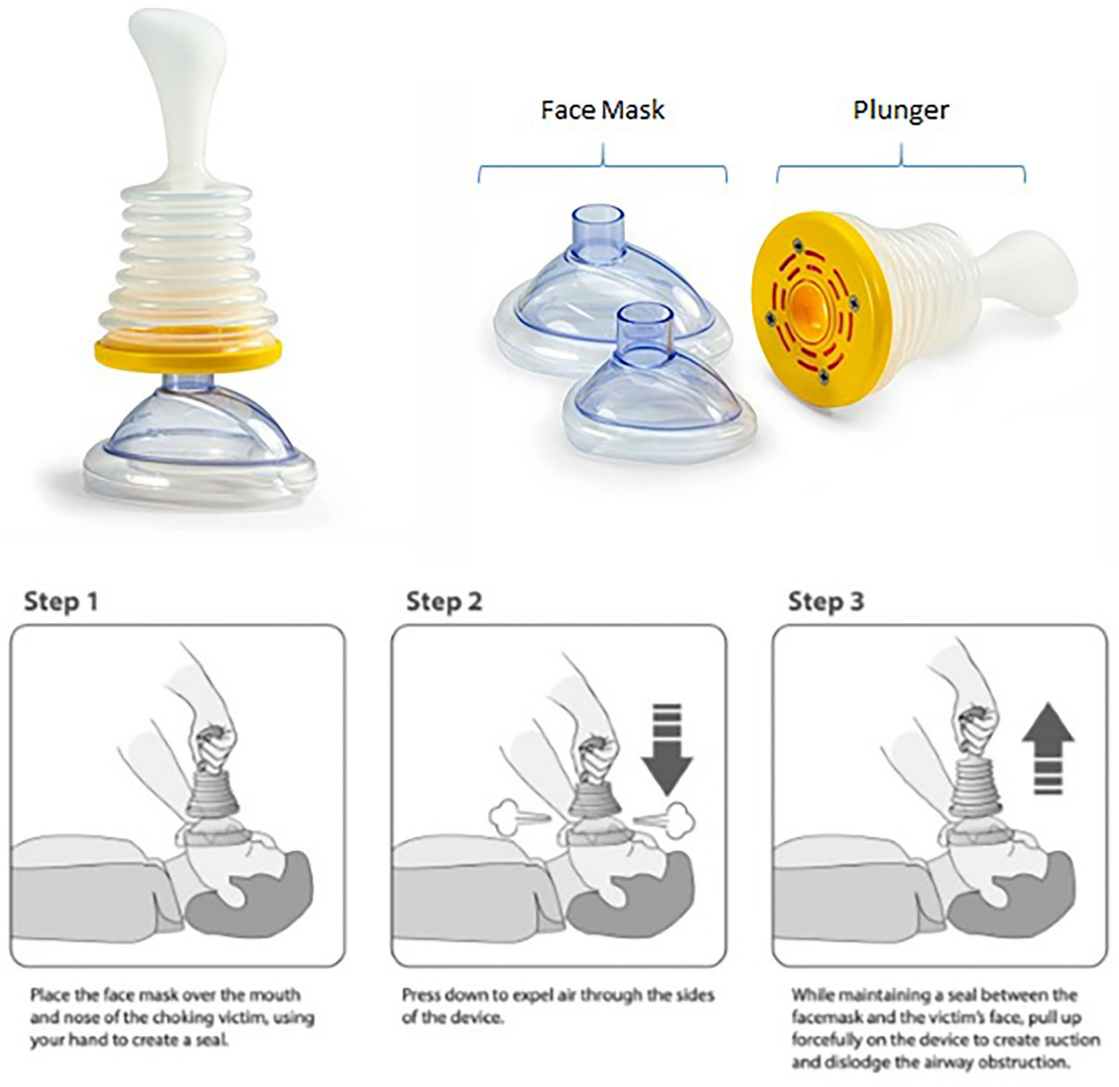
LifeVac device and usage.

In the case of a choking emergency, defined as complete airway obstruction, time is of the essence, as brain damage will occur in 5 min and death will occur in several more minutes without oxygen (4). In the United States alone, 5,051 deaths from choking were reported in 2015 (5). In 1974, an abdominal thrust-based maneuver was developed to remove a bolus of food or other foreign bodies that become trapped in the back of the throat or trachea and obstruct the airway (6). The maneuver relies on forcing the obstruction out of the airway by applying upward thrusts to the epigastrium. The current American Heart Association choking protocol described back blows and abdominal thrusts for resuscitation of an adult choking victim, with a progression to chest thrusts if the abdominal thrusts are not effective (7). Current protocols suggest cardiopulmonary resuscitation (CPR) if abdominal thrusts do not provide a resolution to the choking incident which, without a patent airway, is likely to be futile as well as hazardous in that the object may be forced further into the airway by rescue breaths. In addition, maneuvers such as back blows and abdominal thrusts become almost impossible in individuals who are wheelchair bound, pregnant, or morbidly obese. While the use of Magill forceps has proven successful in choking cases refractory to abdominal thrusts, this is an invasive and more advanced skill that cannot be employed by an untrained caregiver (8). If a choking incident cannot be resolved by persons on-scene, emergency medical services (EMS) can be called to intervene. However, the average time for emergency responders to arrive on the scene of an emergency after a 911 call is placed is 7 min to as long as 14 min in the rural setting (9), making it unlikely that they will arrive before brain damage has occurred. Until recently a non-invasive device that could be used by both laypersons and medical professionals to assist in a choking emergency when standard maneuvers fail did not exist. A novel, non-powered suction device for resuscitation of a choking victim has been developed (LifeVac LLC, Nesconset, NY; Figure 1). The device is FDA registered and has been available since 2014. Over 80,000 units have been distributed worldwide, including to the United Kingdom, Greece, United States, Australia, Israel, and Spain (LifeVac LLC data). This simple-to-use, lightweight, portable, non-powered suction device includes a plunger with a patented one-way valve such that when the plunger is depressed, air is forced out the sides and not into the victim, and when the plunger is pulled back, suction is applied. The device attaches to a standard facemask, creating a seal over the nose, and mouth. Upon pulling up on the plunger, the object is removed from the airway (Figure 1). This case series summarizes user-reported implementations of the device in patients with oropharyngeal dysphagia during choking emergencies.

## Methods

Each device is supplied with either a feedback card that can be mailed to the company, or a card that directs the user to a website form such that if the unit is utilized the user can provide feedback regarding the event, including any complications encountered (10). The user can also request a free replacement of the device after deployment using this form, as it is a single use device. The use of the device is intuitive and when the use has been assessed in non-clinical lay people, the simplicity of its use has been confirmed. The device is shipped with both an online training video and explicit written directions as well as a practice mask so the user can practice upon receiving and become comfortable with its use (11). As part of an internal monitoring study, the manufacturer of the device has kept track of all reported uses of the device. Reports of use in patients with no underlying conditions causing oropharyngeal dysphagia were excluded. A subset of preliminary data was presented as a poster at The World Congress of Gastroenterology at the American College of Gastroenterology in October 2017, and reported as case studies (12, 13). Data that summarize the resuscitation of pediatric choking victims, as defined by an individual suffering from a complete airway obstruction, using this device was recently published (14).

## Results

Between January 2014 and July 2020 there were no reported failures of the device. A total of 42 reports of use on adult choking emergencies have been documented, 39 of which included patients with conditions predisposing them to oropharyngeal dysphagia, specifically advanced age (over 80 years old), cerebral palsy, dementia (including Alzheimer's disease), Down syndrome, Huntington's disease, multiple sclerosis, neurodegenerative disease, non-specific Parkinson's disease, severe intellectual disability, spina bifida, stroke, and traumatic brain injury. Further demographics are summarized and reviewed in Table 1. The majority of the patients resided in European countries (*n* = 32), with six in the United States of America, and one from Australia. Ten had no predisposing conditions besides advanced age, but the majority of the patients had a medical condition that predisposed them to oropharyngeal dysphagia. Ten of the patients were wheelchair-bound, making abdominal thrusts difficult. Another patient was described as “too frail for abdominal thrusts,” while one patient had a percutaneous gastrostomy, making abdominal thrusts impossible.

**Table 1:** Summary of 39 cases with risk factors for oropharyngeal dysphagia.

**Characteristic**	**Value**
Age range, years	28–98
Sex, *n*	
Male	18
Female	18
Not reported	3
Medical condition, *n*	
Advanced age	10
Cerebral palsy	5
Dementia (including Alzheimer's disease)	7
Down syndrome	2
Huntington's disease	2
Multiple sclerosis	2
Neurodegenerative disease, nonspecific	3
Parkinson's disease	3
Severe intellectual disability	1
Spina bifida	1
Stroke	2
Traumatic brain injury	1
Geographical location, *n*	
Europe	32
United States of America	6
Australia	1
Location of event, *n*	
Care home	33
Home/Car	2
Unknown	4
Person using device, *n*	
Nurse/other medical professional	34
Lay person	3
Unknown	2
No. of attempts, *n*	
1	10
2	8
3+	16
Unknown	5
Object removed, *n*	
Apple	1
Bread	4
Burger	1
Chicken	5
Chocolate	1
Coleslaw	1
French fries	1
Meat	3
Melon	1
Mushroom	1
Potato	3
Porridge	1
Rice	1
Saliva/Phlegm	5
Sandwich	1
Sausage	2
Tuna sandwich	1
Unknown	6
Patient consciousness, *n*	
Conscious	17
Unconscious	15
Unknown	7

In 38 patients the device resolved the choking incident and the patients survived. Although the device successfully removed the blockage from the 39^th^ patient, as confirmed by paramedics who arrived on the scene, the patient was unable to be revived despite receiving 20 min of CPR. The device was used multiple times in several patients in order to resolve the choking incident, resulting in a total of at least 100 device implementations. In nine of the reported cases the first application of the device was successful in dislodging the foreign body from the airway and resulted in no adverse events. In the event of multiple applications, each patient returned to baseline health status without further incident, except for Patient 39, who was discussed above.

There were a few occasions where the device partially resolved the choking incident but further medical intervention was needed to fully remove the airway obstruction. In one patient, three attempts partially dislodged a piece of meat so that the patient could move air on his own and achieved SpO_2_ of 100% with supplemental oxygen, but EMS staff suspected that a partial airway obstruction persisted due to the presence of wheezing. After two additional applications by EMS staff, an emergency department physician successfully removed the partial airway obstruction by using the device three times in the hospital. In a patient with Alzheimer's disease who choked on a hamburger multiple device applications were required in both the pre-hospital and hospital setting to remove the boluses; all obstructions were fully removed in the emergency room. Two additional patients required the use of a powered suction device after the non-powered device partially removed their airway obstructions to fully resolve the issue.

The device was used successfully by a variety of individuals including EMS providers, an in-hospital physician, care home staff, and laypersons on conscious and unconscious choking victims. User reports were generally favorable in terms of their experiences employing the device during a choking emergency. Two users reported difficulty forming a seal with the face mask because the patients were diaphoretic. In the case of excessive sweatiness or other secretions present around the victim's mouth, users should take care to wipe the victim's face to help facilitate a better seal. No serious adverse events were reported. One user remarked that the face mask left a contusion on the patient's nasal bridge, but since a further update was not received it's assumed the trauma resolved without further intervention.

## Discussion

In the event of a choking emergency current choking protocols suggest back blows and abdominal thrusts with a progression to chest compressions if abdominal thrusts do not dislodge the airway obstruction (7). While these protocols have been proven to be successful 86% of the time, they can result in complications (8, 15). Morbid obesity, pregnancy, and being wheelchair-bound can prevent the successful administration of standard anti-choking maneuvers. Additionally, when these maneuvers fail, one is left waiting for emergency personnel or continuing a protocol that has been unsuccessful thus far. Invasive procedures, such as a cricothyrotomy or the use of Magill forceps, require advanced medical training and can lead to complications. Therefore, there is an urgent need for an inexpensive, readily available, simple-to-use resuscitation aid for use during a choking emergency. A novel portable non-invasive suction device has been developed, which may have significant utility during a choking emergency.

The strengths of this study is the independent analysis of self-reported data regarding the experience with a novel portable non-invasive suction device. As all reported uses of the device in people with underlying oropharyngeal predisposing risks were included, there was no opportunity for bias in summarizing these outcomes. This device has been reported to be successful in more than 70 real-life choking emergencies worldwide (16). No significant adverse events have been reported thus far. While there may be concerns over esophageal or pulmonary injury from the force generated with this device, no barotrauma related injuries were reported to date.

The limitations of this study are that this was a small, retrospective report of events that occurred and was not a prospective randomized study. However, it is impossible to design an ethical controlled prospective randomized clinical trial of the device in live human subjects to demonstrate efficacy. No suitable animal model that simulates human facial structure is available for study. A study in a human cadaver found that the device successfully removed simulated food boluses of varying sizes 49/50 times (17). The device has also demonstrated efficacy when used on a choking simulator mannequin (18). There have been no reports of failure of the device; although Patient 39 was not resuscitated, the device did successfully remove the obstruction, as confirmed by paramedics who assessed and treated the patient on-scene. However, since this current report relies on self-reported accounts of device use we cannot definitively state that no failures or complications have occurred, since it is not mandatory for users to report their experiences. While there is a training video available online (11), there is no way to determine whether the individuals completed any training prior to device utilization, and whether the device was used correctly in each event. However, given the promising real-world data reported thus far, the device deserves further consideration and study in patients with oropharyngeal dysphagia who are at increased risk of choking.

## Data Availability Statement

The original contributions presented in the study are included in the article/supplementary material, further inquiries can be directed to the corresponding author/s.

## Ethics Statement

Ethical review and approval was not required for the study on human participants in accordance with the local legislation and institutional requirements. Written informed consent for participation was not required for this study in accordance with the national legislation and the institutional requirements. An IRB waiver was obtained on the basis of the above.

## Author Contributions

All authors listed have made a substantial, direct, and intellectual contribution to the work and approved it for publication.

## Conflict of Interest

The authors declare that the research was conducted in the absence of any commercial or financial relationships that could be construed as a potential conflict of interest.

## Publisher's Note

All claims expressed in this article are solely those of the authors and do not necessarily represent those of their affiliated organizations, or those of the publisher, the editors and the reviewers. Any product that may be evaluated in this article, or claim that may be made by its manufacturer, is not guaranteed or endorsed by the publisher.
